# A brain-like classification method for computed tomography images based on adaptive feature matching dual-source domain heterogeneous transfer learning

**DOI:** 10.3389/fnhum.2022.1019564

**Published:** 2022-10-11

**Authors:** Yehang Chen, Xiangmeng Chen

**Affiliations:** ^1^Laboratory of Artificial Intelligence of Biomedicine, Guilin University of Aerospace Technology, Guilin, China; ^2^School of Electronic Information and Automation, Guilin University of Aerospace Technology, Guilin, China; ^3^Department of Radiology, Jiangmen Central Hospital, Jiangmen, China

**Keywords:** solitary pulmonary solid nodule, heterogeneous transfer learning, adaptive feature matching, extreme learning machine, sparse Bayesian, ensemble learning

## Abstract

Transfer learning can improve the robustness of deep learning in the case of small samples. However, when the semantic difference between the source domain data and the target domain data is large, transfer learning easily introduces redundant features and leads to negative transfer. According the mechanism of the human brain focusing on effective features while ignoring redundant features in recognition tasks, a brain-like classification method based on adaptive feature matching dual-source domain heterogeneous transfer learning is proposed for the preoperative aided diagnosis of lung granuloma and lung adenocarcinoma for patients with solitary pulmonary solid nodule in the case of small samples. The method includes two parts: (1) feature extraction and (2) feature classification. In the feature extraction part, first, By simulating the feature selection mechanism of the human brain in the process of drawing inferences about other cases from one instance, an adaptive selected-based dual-source domain feature matching network is proposed to determine the matching weight of each pair of feature maps and each pair of convolution layers between the two source networks and the target network, respectively. These two weights can, respectively, adaptive select the features in the source network that are conducive to the learning of the target task, and the destination of feature transfer to improve the robustness of the target network. Meanwhile, a target network based on diverse branch block is proposed, which made the target network have different receptive fields and complex paths to further improve the feature expression ability of the target network. Second, the convolution kernel of the target network is used as the feature extractor to extract features. In the feature classification part, an ensemble classifier based on sparse Bayesian extreme learning machine is proposed that can automatically decide how to combine the output of base classifiers to improve the classification performance. Finally, the experimental results (the AUCs were 0.9542 and 0.9356, respectively) on the data of two center data show that this method can provide a better diagnostic reference for doctors.

## Introduction

With the development of computed tomography (CT) technology, the detection rate of solitary pulmonary solid nodule (SPSN) has greatly increased (Henschke et al., 2018). Lung granuloma (LG) is a typical histopathological manifestation of benign SPSN. Lung adenocarcinoma (LA) is the most common histological subtype of primary lung cancer (Travis et al., 2011). In clinical practice, the malignancy risk degree must be assessed to determine the appropriate treatment plan when an SPSN is found. More aggressive treatment options are recommended to improve prognosis for LA patients. Conversely, LG patients should avoid unnecessary treatment procedures (such as surgery and chemotherapy). However, LG is similar to LA in SPSN patients in terms of CT images, which creates a diagnostic dilemma for clinicians (Starnes et al., 2011; Boskovic et al., 2014; Sollini et al., 2017). Therefore, it is necessary to develop an accurate and efficient method for the preoperative differentiation of LG and LA in SPSN patients.

Convolutional neural network (CNN) is widely used in medical image research. It simulates the mechanism of human brain to interpret data, that is, by building a hierarchical model structure similar to human brain to step by step extracts effective features directly related to tasks the bottom to the top level of from input data (Shin et al., 2016; Interian et al., 2018; Tan et al., 2018). However, the size of datasets in the medical field is often small, and CNN is prone to over-fitting under the condition of small samples. To improve the effect of CNN under small samples, scholars have introduced transfer learning into CNN (Tan et al., 2018; Feng et al., 2021). By imitating the mechanism of drawing inferences about other cases from one instance of the human brain, transfer learning uses the knowledge learned from a large source domain data to help the learning of the target task. In medical imaging studies of pulmonary nodules, model-based fine-tuning is a commonly used transfer learning strategy. First, a source network is trained on a large dataset (such as ImageNet). Then, the learned weights are used as the initial weight of the target network. Finally, the target network is fine-tuned by using the target data (Zhao et al., 2018; Harsono et al., 2020). However, when there is a large semantic difference between source domain data and target domain data, the transfer learning based on fine-tuning will still overfit (Romero et al., 2020; Liu et al., 2021).

To this end, heterogeneous transfer learning is proposed by scholars. In heterogeneous transfer learning, features can be transferred between different domains by feature matching. Romero et al. proposed a teacher-student training model to transfer knowledge from the deeper teacher network to the shallower student network by calculating the matching loss between teacher features and student features (Romero et al., 2014). (Zagoruyko and Komodakis, 2018) and (Srinivas and Fleuret, 2018) proposed attention transfer and Jacobian matrix matching methods, and realized knowledge transfer by using a feature map or Jacobian matrix to generate attention attempts, respectively. Although the above methods make the model have certain effects in the case of heterogeneous data sources, there are still two problems: (1) they cannot adaptive determine the importance of the features of the source network relative to the target task, and negative transfer may occur when redundant features in the source network are transferred to the target network (Zeiler and Fergus, 2014). (2) They only empirically determine how the features in the source network are transferred to the target network, which will consume many human and material resources, and the results may not be optimal.

In addition, most of the existing transfer learning research works were based on the knowledge transfer of a single source domain, namely, single-source transfer learning. In the field of medical imaging, ImageNet is generally used as the source domain data of transfer learning because the network trained by ImageNet has rich basic texture information. For example, Nobrega et al. used Imagenet dataset to train ResNet 50 as a feature extractor, then used LIDC dataset to extract features, and finally used SVM-RBF to classify them (Nóbrega et al., 2018). Buty et al. used Imagenet data set to pre-train ResNet 50, and LIDC data set to fine tune the pre training model and serve as a feature extractor. Finally, combined with shape features, random forest classifier was used to estimate the malignant degree of pulmonary nodules (Buty et al., 2016). Wang et al. used Imagenet datasetto pre train the Alexnet network, then extracted the deep learning features of the region of interest through transfer learning, combined with manual features to form combined features, and used random forest to classify them, which improved the classification accuracy of pulmonary nodules to a certain extent (Wang et al., 2016). However, for the target domain, the single source domain cannot provide rich and multiview knowledge; that is, single-source domain transfer learning has the problem of insufficient information (Zhang et al., 2016; Luo et al., 2018). Transfer learning using multiple source domain data is a solution. It can provide various knowledge to help the learning of the target task. In medical imaging, medical images of the same tissue (e.g., whole slide images (WSIs) and CT images of the lung) can be related. WSIs of the lung can provide a large amount of microscopic information about the tumor under the microscope. In contrast, lung CT images can reflect tumor imaging information at a macro scale. Therefore, knowledge transfer using ImageNet and lung WSI construction source networks at the same time will be more conducive to the training of lung CT image-based target networks. At the same time, in the face of transfer learning of multiple source domains, how to fully exploit the multi-view knowledge provided by these source domains and effectively transfer this knowledge to the target domain is the key to improving the learning performance of the target domain.

Based on this, according the mechanism of the human brain focusing on effective knowledges while ignoring redundant knowledges in recognition tasks, this paper proposes a brain-like classification method for CT images based on adaptive feature matching dual-source domain heterogeneous transfer learning to preoperatively aid in the diagnosis of LG and LA for SPSN patients. This method consists of two parts: feature extraction of adaptive feature matching-based dual-source domain heterogeneous transfer learning and feature classification of an ensemble classifier based on sparse Bayesian extreme learning machine (ELM). First, By simulating the feature selection mechanism of the human brain in the process of drawing inferences about other cases from one instance, an adaptive selection-based dual-source domain feature matching network was proposed to determine the matching weight of each pair of feature maps and each pair of convolution layers between the source network (ImageNet-based source network 1 and lung WSI-based source network 2) and the target network, respectively. These two weights can, respectively, automatically select the features of the source network conducive to target task learning and the destination of feature transfer to restrict the training of the target network and improve the robustness of the target network. Meanwhile, a target network based on diverse branch block was proposed that made the target network have different receptive fields and complexity paths to further improve the feature expression ability of the target network. After training the target network, the diverse branch block was equivalently converted into a convolution kernel, which will make the target network not only have rich feature expression ability but also reduces the inference time cost. Then, the Convolution kernel after reparameterization of the target network was used as the feature extractor to extract the features. In addition, the clinical features and CT findings were included in the analysis to carry out a comprehensive analysis of the patients. Finally, an ensemble classifier based on sparse Bayes ELM was proposed. Ensemble learning can automatically bias how to combine the output of different base classifiers to improve classification performance.

## Materials and methods

### Research data

The data of 684 SPSN patients from two medical centers were collected. These patients were diagnosed with LA or LG by surgical histopathology. CT images within 4 weeks before surgery, clinical features and CT findings of the SPSN patients were obtained. Details of the SPSN patients are shown in Table 1. The training cohort included 260 patients (105 LG and 155 LA) from medical center 1. Test cohort 1 included 216 patients (79 LG and 137 LA) from medical center 1. Test cohort 2 included 208 patients (57 LG and 151 LA) from medical center 2. The clinical features included gender and age. The CT findings, such as lesion size, spiculation sign, lobulated sign, and shape sign, were obtained by radiologists based on the CT images. The effects of the above clinical features and CT findings for LA and LG diagnosis of SPSN patients were confirmed by clinical studies (El-Baz et al., 2010; Dhara et al., 2016; MacMahon et al., 2017; Feng et al., 2019).

**TABLE 1:** Information of SPSN patients in this study.

Clinical features and CT findings	Training cohort (*N* = 260)		Test cohort 1 (*N* = 216)		Test cohort 2 (*N* = 208)	
			
	LG (*N* = 105)	LA (*N* = 155)	LG (*N* = 79)	LA (*N* = 137)	LG (*N* = 57)	LA (*N* = 151)
**Gender**						
Male	69	65	50	75	33	61
Female	36	90	29	62	24	90
**Age (mean ± SD, year)**	51.87 ± 12.32	60.87 ± 10.04	51.03 ± 12.85	61.01 ± 9.96	56.74 ± 13.08	59.99 ± 10.48
**Lesion size (mean ± SD, mm)**	12.10 ± 7.10	17.56 ± 7.73	12.43 ± 6.55	17.57 ± 7.43	12.93 ± 7.33	21.03 ± 8.45
**Shape sign**						
Regular	50	20	31	6	23	10
Irregular	55	135	48	131	34	141
**Lobulated sign**						
Absence	59	26	43	11	37	14
Presence	46	129	36	126	20	137
**Spiculation sign**						
Absence	67	75	54	80	49	66
Presence	38	80	25	57	8	85

The CT images were obtained from a dual-energy Somatom Flash and 64-detector-row Aquilion One CT scanner. The CT scanning scheme was as follows: tube voltage of 120 kVp; the tube current was automatically adjusted by the patient’s body weight; spiral mode with collimation of 16 × 0.75 mm or 64 × 0.5 mm and pitch of 0.875-1.5; slice thickness 1.0-3.0 mm; and slice interval 0.8-3.0 mm. The patients were positioned supine and scanned in the caudocranial direction. The scanning included imaging from the thoracic inlet to the bilateral adrenal glands with deep inspiration breath-hold. The CT images were analyzed in the lung window (window width: 1,500 Hounsfield Unit (HU) and window level: −600 HU).

In addition, the WSIs of lung cancer from the Cancer Genome Atlas (TCGA) and nature images from ImageNet were collected as the source domain data of transfer learning.

### Data preprocessing

**FIGURE 1:**
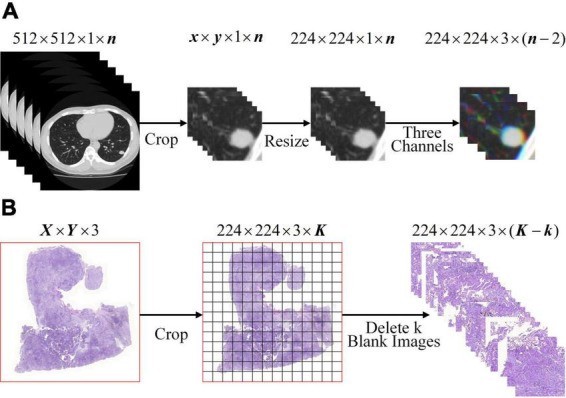
Image preprocessing. **(A)** CT image preprocessing of SPSN. **(B)** WSIs preprocessing. Notice: *K* =[*^X^*/_224_] × [*^Y^*/_224_].

To meet the input of the network, the lung WSIs from TCGA and CT images of SPSN were preprocessed into 224×224×3 three-channel images on the basis that the lesions were completely surrounded. For CT images: as showed in Figure 1A, firstly, the CT images were cropped by finding a rectangular region of interest that enclosed the outline of SPSN. Secondly, the region of interest was resized to 224 by 224 square. Thirdly, a series of three channel images which were composed of three consecutive slices. For WSIs: as showed in Figure 1B, firstly, each WSI is divided into a plurality of small images with a size of 224 by 224. At this time, some of the small images were blank. Secondly, the blank images were discarded to eliminate its influence on the model training. After preprocessing, 285, 994 lung squamous cell carcinoma WSIs and 290,554 lung adenocarcinoma WSIs were obtained. And that, 2, 135 CT images of LA and 630 CT images of LG were obtained in the training cohort.

### Proposed approach

To solve the problem of aided diagnosis of SPSN in the case of small samples, a brain-like classification method based on adaptive feature matching dual-source domain heterogeneous transfer learning was introduced. The proposed method was divided into two components: feature extraction and classification. For a given input image, first, the features were extracted by an adaptive feature matching-based dual-source domain heterogeneous transfer learning model, and then they were classified into one of the classes by an ensemble classifier based on sparse Bayesian ELM.

#### Feature extraction

**FIGURE 2:**
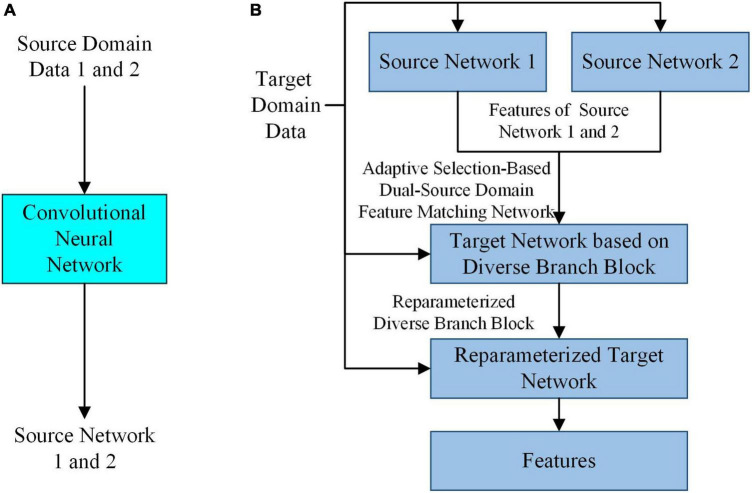
The overall architecture of the feature extraction model. **(A)** Training of the source network. **(B)** Training of the target network based on diverse branch block and feature extraction.

In the case of small samples, to extract features that can accurately reflect the intrinsic properties of SPSN and have high robustness, by simulating the feature selection mechanism of the human brain in the process of drawing inferences about other cases from one instance, a feature extraction model based on adaptive feature matching dual-source domain heterogeneous transfer learning was proposed. As shown in Figure 2, the adaptive selection-based dual-source domain feature matching network in the feature extraction model can select features from the source network that are helpful for the training of the target task. It can constrain the training of the target network to avoid the influence of redundant features in the source network on the target network. In addition, a diverse branch block was introduced into the target network to further enhance the feature expression ability of the convolution kernels.

##### Adaptive selection-based dual-source domain feature matching network

**FIGURE 3:**
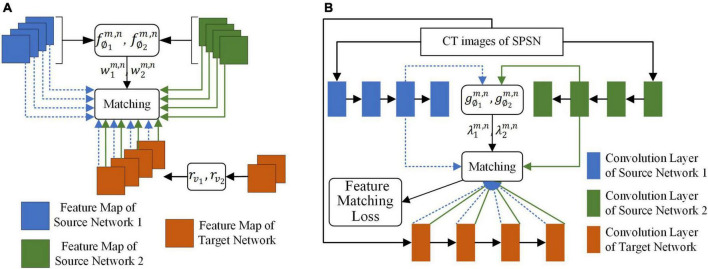
The adaptive selection-based dual-source domain feature matching network for selective knowledge transfer. **(A)** Matching of the feature map between the source networks and the target network. **(B)** Matching of the feature map of the convolution layer between the source networks and the target network.

The goal of the adaptive selection-based dual-source domain feature matching network was to select features in the source network that were beneficial to the target task to constrain the training of the target network without manual association to select features. As shown in Figure 3, given the source network and target network, the adaptive selection-based dual-source domain feature matching network determines (1) what features in the source network should be transferred and the weight of the transfer and (2) how the valuable features of the source network should be transferred to the target network.

To achieve the above goals, a feature match item should be defined. Let *x* be the input of the network, $S_{k}^{m}\,\left(x\right)$ be the feature map of the mth layer of the kth source network, where *k* ∈ 1,2…, and $T_{\theta }^{n}\,\left(x\right)$ be the feature map of the nth layer of the target network with parameterθ. To achieve the goals of the adaptive selection-based dual-source domain feature matching network, a *l*_2_ norm distance between $S_{k}^{m}\,\left(x\right)$ and $T_{\theta }^{n}\,\left(x\right)$ was designed. The features that were beneficial for learning the target task were selected by minimizing the *l*_2_ norm distance. This *l*_2_ norm distance was defined as


(1) $$l_{2}\,\mathrm{norm}\,\mathrm{distance}=\sum_{k=1}^{2}{∥r_{\upsilon_{k}}\,\left(T_{\theta }^{n}\,\left(x\right)\right)-S_{k}^{m}\,\left(x\right)∥}_{2}^{2}$$

For each source network, a linear transformation _*r*υ_*k*__ was set for the target network, such as a pointwise convolution, to ensure that $T_{\theta }^{n}\,\left(x\right)$ had the same number of channels as $S_{k}^{m}\,\left(x\right)$. _*r*υ_*k*__ was necessary for target network training but not for testing. υ_*k*_ represented the parameter of the linear transformation of the kth source network with respect to the target network. To minimize Equation 1, we had to obtain two matching losses.

First, in transfer learning, redundant features in the source network may cause negative transfer of the target network. To give more attention to the feature maps that are beneficial to target task learning, a weighted feature matching loss was designed for each feature map in the source network. For the kth source network, this loss was defined as


$$L_{w\,f\,m_{k}}^{m,n}\left(\theta ,\upsilon_{k}|x,w_{k}^{m,n}\right)=\frac{1}{H\,W}\sum_{c_{k}}w_{c_{k}}^{m,n}\sum_{i,j}(r_{\upsilon_{k}}{\left(T_{\theta }^{n}\left(x\right)\right)}_{c_{k},i,j}$$

(2) $$-S_{k}^{m}{\left(x\right)}_{c_{k},i,j}){}^{2}$$

where *H*×*W* is the feature map size of $S_{k}^{m}\,\left(x\right)$ and $r_{\upsilon_{k}}\,\left(T_{\theta }^{n}\,\left(x\right)\right)$. The value range of *i* was 1 to H, and the value range of *j* was 1 to W. *c*_*k*_ indicates the cth channel of the kth source network. $w_{c_{k}}^{m,n}$ is a learnable weight, which reflects the transferability of the feature map of channel c of $S_{k}^{m}\,\left(x\right)$ relative to $r_{\upsilon_{k}}\,\left(T_{\theta }^{n}\,\left(x\right)\right)$, and $\sum_{c_{k}}w_{c_{k}}^{m,n}=1,w_{c_{k}}^{m,n}>0$. The single-layer fully connected neural network $f_{\emptyset_{k}}^{m,n}$ is designed to learn $w_{c_{k}}^{m,n}$. ∅_*k*_ represented parameter of the *f^m^*^,*n*^ the kth source network with respect to the target network. The input of $f_{\emptyset_{k}}^{m,n}$ is the global mean pooling of each feature map in $S_{k}^{m}\,\left(x\right)$, and the output of $f_{\emptyset_{k}}^{m,n}$ is the softmax form. That is,


(3) $$w_{c_{k}}^{m,n}=f_{\emptyset_{k}}^{m,n}\,\left(S_{k}^{m}\,\left(x\right)\right)$$

where ∅_*k*_ is the parameter of $f_{\emptyset_{k}}^{m,n}$. As shown in Figure 3A, for a given $S_{k}^{m}\,\left(x\right)$ and $r_{\upsilon_{k}}\,\left(T_{\theta }^{n}\,\left(x\right)\right)$, in each input image, different weights were given to different feature maps in $S_{k}^{m}\,\left(x\right)$, and the more important feature maps correspond to the larger weights, which makes the loss corresponding to the feature map receive more attention.

Second, after we know which features in the source network should be transferred to the target network, we also need to know where these features should be transferred to the target network. As shown in Figure 3B, the output of each convolution layer of $S_{k}^{m}\,\left(x\right)$ or $r_{\upsilon_{k}}\,\left(T_{\theta }^{n}\,\left(x\right)\right)$ was used as a unit. For each pair of convolution layers (*m*,*n*), a learnable parameter $\lambda_{k}^{m,n}>0$ was introduced to express the degree of transferability of features between $S_{k}^{m}\,\left(x\right)$ and $r_{\upsilon_{k}}\,\left(T_{\theta }^{n}\,\left(x\right)\right)$. A larger $\lambda_{k}^{m,n}$ indicated that the features of the pair of convolution layers were more beneficial to the learning of the target task. Similarly, a single-layer fully connected neural network $g_{\Phi_{k}}^{m,n}$ was designed to learn the value of $\lambda_{k}^{m,n}$ to adaptive select the matching pair of the convolution layer of the source network to the convolution layer of the target network. Φ_*k*_ represented parameter of the *g^m^*^,*n*^ the kth source network with respect to the target network. The global mean pooling of each convolution layer of $S_{k}^{m}\,\left(x\right)$ was used as the input of $g_{\Phi_{k}}^{m,n}$. The output of $S_{k}^{m}\,\left(x\right)$ was given in the form of ReLU6 to ensure nonnegativity of $\lambda_{k}^{m,n}$ and prevent $\lambda_{k}^{m,n}$ from becoming too large. That is,


(4) $$\lambda_{k}^{m,n}=g_{\Phi_{k}}^{m,n}\,\left(S_{k}^{m}\,\left(x\right)\right)$$

After the weight $w_{c_{k}}^{m,n}$ of each pair of feature maps of $S_{k}^{m}\,\left(x\right)$ relative to $r_{\upsilon_{k}}\,\left(T_{\theta }^{n}\,\left(x\right)\right)$ and the weight $\lambda_{k}^{m,n}$ of each convolution layer pair were obtained, the loss of the adaptive selection-based dual-source domain feature matching network can be defined as


$$L_{w\,f\,m}\,\left(\theta ,\upsilon |x,\phi ,\Phi \right)=\sum_{k=1}^{2}\sum_{\left(m,n\in p_{k}\right)}\lambda_{k}^{m,n}\,L_{w\,f\,m_{k}}^{m,n}$$

(5) $$\left(\theta ,\upsilon_{k}|x,w_{k}^{m,n}\right)$$

where *P*_*k*_ is the set of candidate convolution layer pairs of $S_{k}^{m}\,\left(x\right)$ and $r_{\upsilon_{k}}\,\left(T_{\theta }^{n}\,\left(x\right)\right)$. ϕ ∈ (ϕ_1_,ϕ_2_), Φ ∈ (Φ_1_,Φ_2_) and υ ∈ (υ_1_,υ_2_). Then, the final loss function of the adaptive feature matching-based dual-source domain heterogeneous transfer learning model was defined as


(6) $$L_{t\,o\,t\,a\,l}\,\left(\theta ,\upsilon |x,y,\phi ,\Phi \right)=L_{o\,r\,g}\,\left(\theta |x,y\right)+\varsigma \,L_{w\,f\,m}\,\left(\theta ,\upsilon |x,\phi ,\Phi \right)$$

where *L*_*org*_(θ|*x*,*y*) is the original loss of the target network and ς is a hyper-parameter.

##### Target network based on diverse branch block

**FIGURE 4:**
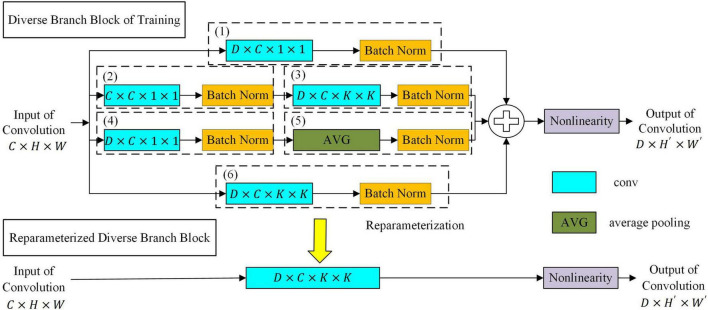
The diverse branch block structure.

To further improve the feature expression ability of the target network, a convolution neural network based on diverse branch block was proposed as the target network. As shown in Figure 4, the network replaces a convolution kernel with a diverse branch block. The target network based on diverse branch block had different receptive fields and paths of different complexities by combining branch structures of different scales and complexities (including multi-scale convolution sequences, concatenated convolutions and average pooling) and improved the feature expression ability of the network. The target network based on diverse branch block was used during target network training. After the target network was trained, the diverse branch block was equivalently converted into a single convolution kernel according to the homogeneity and additivity of convolution. At this time, the equivalent transformed network structure is used during verification/inference, which will make the target network not only have rich feature expression ability but also reduces the inference time cost (Ding et al., 2021).

Let the data of the input of the convolution kernel be *I* ∈ ℝ*^C^*^×*H*×*W*^. *C* is the number of inputs of feature maps. *H*×*W* is the size of the feature map. The parameters of the convolution kernel are *F* ∈ ℝ*^D^*^×*C*×*K*×*K*^, where D is the number of output channels and *K*×*K* is the size of the convolution kernel. The output of the convolution kernel is *O* ∈ ℝ*^D^*^×*H*′×*W*′^. *H*′×*W*′ is the size of the output feature map, which is determined by the settings of *K*, padding, and stride. We use ⊗ to denote the convolution operator and formulate the bias addition by replicating the bias *b* into *REP*(*b*) ∈ ℝ*^D^*^×*H*′×*W*′^ and adding it to the results of the convolution. According to the homogeneity and additivity of convolution, the equivalent transformation of the diverse branch block into a single convolution kernel is shown below.


(7) $$F_{d,c,k,k}^{\prime }\,\left\{ \begin{array}{c} \begin{array}{c} \frac{\gamma^{\left(1\right)}}{\sigma^{\left(1\right)}}F^{\left(1\right)}+\frac{\gamma^{\left(3\right)}}{\sigma^{\left(3\right)}}F^{\left(3\right)}⊗TRANS\left(F^{\left(2\right)}\right)+\frac{\gamma^{\left(5\right)}}{\sigma^{\left(5\right)}}F^{\left(5\right)}⊗\\ T\,R\,A\,N\,S\,\left(F^{\left(4\right)}\right)+\frac{\gamma^{\left(6\right)}}{\sigma^{\left(6\right)}}\,F^{\left(6\right)}\\ ,ifk=\frac{K+1}{2}andd=c\\ \begin{array}{c} \frac{\gamma^{\left(3\right)}}{\sigma^{\left(3\right)}}\,F^{\left(3\right)}⊗T\,R\,A\,N\,S\,\left(F^{\left(2\right)}\right)+\frac{\gamma^{\left(5\right)}}{K^{2}\,\sigma^{\left(5\right)}}⊗T\,R\,A\,N\,S\,\left(F^{\left(4\right)}\right)\\ +\frac{\gamma^{\left(6\right)}}{\sigma^{\left(6\right)}}\,F^{\left(6\right)}\\ ,ifk\neq \frac{K+1}{2}andd=c \end{array} \end{array}\\ \frac{\gamma^{\left(1\right)}}{\sigma^{\left(1\right)}}\,F^{\left(1\right)}+\frac{\gamma^{\left(3\right)}}{\sigma^{\left(3\right)}}\,F^{\left(3\right)}⊗T\,R\,A\,N\,S\,\left(F^{\left(2\right)}\right)+\frac{\gamma^{\left(6\right)}}{\sigma^{\left(6\right)}}\,F^{\left(6\right)}\\ ,ifk=\frac{K+1}{2}andd\neq c\\ \frac{\gamma^{\left(3\right)}}{\sigma^{\left(3\right)}}\,F^{\left(3\right)}⊗T\,R\,A\,N\,S\,\left(F^{\left(2\right)}\right)+\frac{\gamma^{\left(6\right)}}{\sigma^{\left(6\right)}}\,F^{\left(6\right)}\\ ,ifk\neq \frac{K+1}{2}andd\neq c \end{array}\right.$$

(8) $$\begin{array}{c} \begin{array}{l} REP\left(b^{'}\right)=REP\left({\text{β}}^{\left(1\right)}-\frac{{\text{μ}}^{\left(1\right)}{\text{γ}}^{\left(1\right)}}{\sigma^{\left(1\right)}}\right)+REP\left({\text{β}}^{\left(2\right)}-\frac{{\text{μ}}^{\left(2\right)}{\text{γ}}^{\left(2\right)}}{\sigma^{\left(2\right)}}\right)\\ \;\;\;\;\;⊗F^{\left(3\right)}+REP\left({\text{β}}^{\left(3\right)}-\frac{{\text{μ}}^{\left(3\right)}{\text{γ}}^{\left(3\right)}}{\sigma^{\left(3\right)}}\right)+REP\left({\text{β}}^{\left(4\right)}-\frac{{\text{μ}}^{\left(4\right)}{\text{γ}}^{\left(4\right)}}{\sigma^{\left(4\right)}}\right) \end{array}\\ \;⊗F^{\left(5\right)}+REP\left({\text{β}}^{\left(5\right)}-\frac{{\text{μ}}^{\left(5\right)}{\text{γ}}^{\left(5\right)}}{\sigma^{\left(5\right)}}\right)+REP\left({\text{β}}^{\left(6\right)}-\frac{{\text{μ}}^{\left(6\right)}{\text{γ}}^{\left(6\right)}}{\sigma^{\left(6\right)}}\right) \end{array}$$

where ^μ(*h*)^ ∈ ^ℝ*D*^ and ^σ(*h*)^ ∈ ^ℝ*D*^ are the mean and variance of the batch data of the hth dashed box in Figure 4, respectively. ^γ(*h*)^ ∈ ^ℝ*D*^ and ^β(*h*)^ ∈ ^ℝ*D*^ are the batch-normalized scale factor and bias term of the hth dashed box in Figure 4, respectively.*TRANS*(*F*) is defined as the transpose operation of the convolution, such as *TRANS*(*F^D^*^×*C*×1×1^) = *F^C^*^×*D*×1×1^.

##### Model training strategy

The goal of adaptive feature matching-based dual-source domain heterogeneous transfer learning was to use *L*_*total*_(θ,*v*|*x*,*y*,ϕ,Φ) to train the target network to achieve high performance of the target network. To maximize the performance, the feature matching term *L*_*wfm*_(θ,*v*|*x*,ϕ,Φ) should select features that were beneficial to the learning of the target task. Therefore, a four-stage training method (Jang et al., 2019) was used to jointly train the target network and the feature matching network, thereby alternately updating the target network parameter θ, linear transformation parameter υ, and feature matching network parameter ∅ and Φ.

In the first stage, *L*_*total*_(θ,υ|*x*,*y*,ϕ,Φ) was used to update the target network and linear transformation parameters once. In the second stage, the target network and linear transformation parameters were updated T times by minimizing *L*_*wfm*_(θ,υ|*x*,ϕ,Φ). The target network can be trained by selectively imitating features in the source network that are beneficial to the learning of the target task. More importantly, this increases the influence of the feature matching term *L*_*wfm*_(θ,υ|*x*,ϕ,Φ) on the learning of the target network because the training at this stage only utilizes the knowledge of the source network. In the third stage, the target network and linear transformation parameters were updated one time by minimizing *L*_*org*_(θ|*x*,*y*). In the fourth stage, under the samples used in the first three stages, the speed at which the target network adapts to the target task was measured according to the change in *L*_*org*_(θ|*x*,*y*) from the third stage. Finally, the parameter ∅ and Φ of the feature matching network was updated by minimizing *L*_*org*_(θ|*x*,*y*). The training process iteratively repeats the second to fourth stages until the convergence conditions of the target network are met.

##### Feature extraction of adaptive feature matching-based dual-source domain heterogeneous transfer learning

**FIGURE 5:**
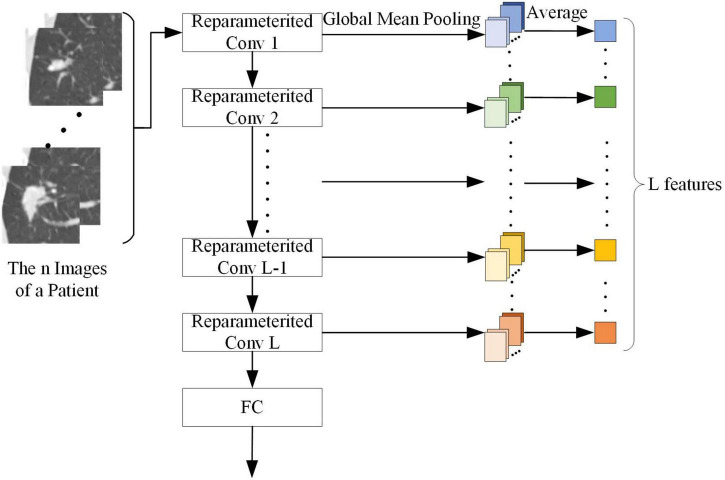
Feature extraction process of a patient.

The output of each convolution kernel in the target network can represent different abstract features of the SPSN. As shown in Figure 5, to make better use of the target network, this paper used the convolution kernel of the reconstructed target network as a feature extractor to extract features. Since each patient has n images with lesions and the features are calculated on a patient basis, after n pictures are processed by a reparameterized convolution kernel and global mean pooling, it is necessary to find an average of n outputs of global mean pooling. The average value is the feature of the reparameterized convolution kernel corresponding to the patient. When the target network has L reparameterized convolution kernels, each patient can extract L features. Then, the Mann-Whitney U test (Ken, 2009) was used to screen out the features of significance in the diagnosis of LG and LA. The Mann-Whitney U test is a nonparametric rank-sum hypothesis test designed to test whether the means of two samples are significantly different. When the *p* value of the Mann-Whitney U test is less than 0.05, it indicates that the feature has a significant effect in the diagnosis of LG and LA.

#### Ensemble classifier based on sparse bayes extreme learning machine

Compared with a single classifier, ensemble learning combining multiple weak classifiers is expected to be a better and more comprehensive strong classifier model (Khellal et al., 2018). Additionally, ELM is a single-hidden layer feedforward neural network, which has the characteristics of a simple structure, fast training speed and high generalization ability (Huang et al., 2006). However, the traditional ELM only considers the training error, which is prone to overfitting in the case of small datasets. In this paper, to prevent overfitting, the *l*_1_ norm was introduced into the ELM to constrain the model so that the model has a sparse solution. However, after introducing the *l*_1_ norm, hyperparameters were inevitably introduced. Therefore, an ensemble classifier based on sparse Bayesian ELM was proposed that can automatically combine the output of the base classifier to improve the performance of the ensemble classifier. The sparse Bayesian not only avoids using time-consuming cross-validation to solve hyperparameters but also has good generalization performance.

**FIGURE 6:**
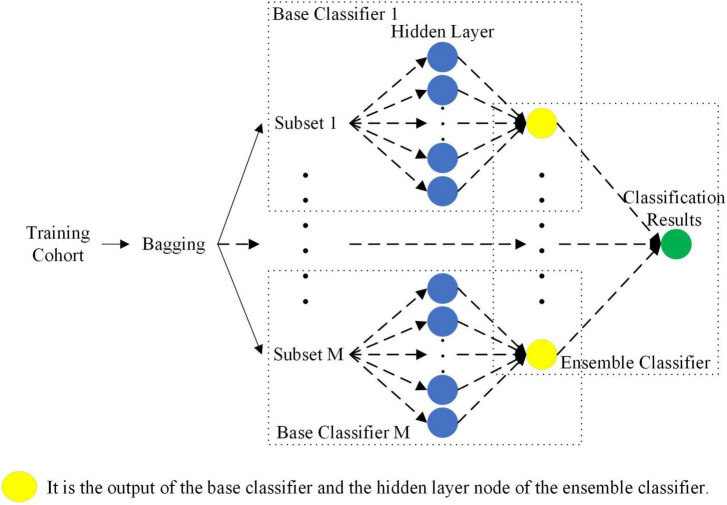
Ensemble classifier based on Sparse Bayes ELM.

As shown in Figure 6, the sparse Bayesian ELM was used as the base classifier and ensemble classifier for ensemble learning. First, the bagging method was used to select the M sample subsets from the training cohort samples. Then, the M base classifiers were trained separately according to these M sample subsets. Finally, the output of the M base classifiers was used as the output of the hidden layer of the ensemble classifier, and the final classification result was calculated. The connection weights between the input layer and the hidden layer of the M base classifiers and the bias of the hidden layer were randomly generated according to the normal distribution. The parameters between the hidden layer and the output layer of the base classifier and the ensemble classifier were solved by sparse Bayes, and the final classification result was obtained.

The objective function of the sparse Bayes ELM was


(9) $$\hat{\text{w}}=a\,r\,g\,m\,i\,n\,{||t-X\,\text{w}-\varepsilon ||}^{2}+\rho \,\sum_{i=1}^{L}{||w_{i}||}_{1}$$

where *t* represents the true label of the sample. w and ε represent the weight and bias between the hidden layer and the output layer, respectively. *w*_*i*_ is the weight between the ith hidden layer nodes and the output node. L is the number of neurons in the hidden layer. ρ > 0 represents the coefficient of the constraint term. *X* represents the output of the hidden layer. When the connection weight between the input layer and the hidden layer and the bias of the hidden layer were determined, X was determined.

For the solution of parameters in Formula (9), this paper proposes a solution method based on sparse Bayesian learning and automatic correlation determination. The Gaussian conjugate sparse prior was introduced into the classical empirical Bayesian linear model to obtain the sparse Bayesian model. That is, it was assumed that bias ε was a zero-mean Gaussian random variable with inverse variance β. Label *t* was modeled as a linear combination with additive Gaussian noise. For the output (X,*t*) of the hidden layer, X ∈ ℝ*^N^*^×*D*^ = ^(_x1_,⋯_x*N*_)*T*^, where N represents the number of samples. The likelihood of the weight vector w can be written as a multivariate Gaussian distribution:


(10) $$p\,\left(t|X,\text{w},\beta \right)=N\,\left(t|X^{T},\text{w},\beta^{-1}\right)$$

To obtain the posterior probability of w, a sparse prior with a multivariate Gaussian distribution with zero mean and diagonal covariance matrix for the weight vector w was introduced (Hoffmann et al., 2008). This sparse prior can be expressed as


(11) $$p\,\left(w|\alpha \right)=\prod_{i=1}^{D}N\,\left(w_{i}|0,\alpha_{i}^{-1}\right)$$

Formula (11) shows that the sparse prior sets a separate hyperparameter α_*i*_ for each weight vector *w*_*i*_, thereby generating a hyperparameter vector α = ^(α_*i*_,⋯α_*D*_)*T*^, which is the diagonal element of the w covariance matrix. Due to the conjugation of the Gaussian prior to the Gaussian likelihood (relative to the mean), the posterior probability is known to be a closed-form Gaussian solution (Hoffmann et al., 2008). The posterior probability of w can be expressed as


(12) p⁢(w|t,X,α,β)=N⁢(w|m,Σ)

The mean m and covariance Σ of the posterior probability distribution of w were defined as


(13) $$\text{m}=\beta \,\text{Σ}\,X^{T}\,t$$

(14) $${\text{Σ}}^{-1}=A+\beta \,X^{T}\,X$$

where = diag(α). The hyperparameters α and β can be calculated by the maximum marginal likelihood method. The marginal likelihood *p*(*t*|α,β) was obtained by integrating the output weight w:


(15) p⁢(t|α,β)=∫⁢p⁢(t|w,β)⁢p⁢(w|α)⁢d⁢w

Then, the log-likelihood can be obtained by squaring the exponent and using the standard form of the normalized coefficient of the Gaussian function:


(16) $$\text{ln}\,p\,\left(t|\alpha ,\beta \right)=\frac{\begin{array}{c} \sum_{i=1}^{D}\text{ln}\,\alpha_{i}+N\,\text{ln}\,\beta +\text{ln}\,\left|\text{Σ}\right|-\\ \beta \,{||\text{t-Xm}||}^{2}-{\text{m}}^{T}\,A\,\text{m}-N\,\text{ln}\,\left(2\,\pi \right) \end{array}}{2}$$

By setting the partial derivatives of the log-likelihood with respect to the hyperparameters α and β to zero, a maximum likelihood estimate of the hyperparameters can be obtained, which is


(17) $$\alpha_{i}^{n\,e\,w}=\frac{\gamma_{i}}{{\text{m}}_{i}^{T}\,A\,{\text{m}}_{i}}$$

(18) $${\left(\beta^{n\,e\,w}\right)}^{-1}=\frac{{||t-X\,\text{m}||}^{2}}{N-\sum_{i=1}^{D}\gamma_{i}}$$

where _m*i*_ is the ith component of the posterior mean m, and γ_*i*_ is defined as


(19) $$\gamma_{i}=1-\alpha_{i}\,{\text{Σ}}_{i\,i},i\in \left\{1,2,\cdots ,D\right\}$$

where Σ_*ii*_ is the ith diagonal component of the posterior distribution covariance Σ . In autocorrelation determination, some elements in α tend to infinity when maximizing the marginal likelihood with respect to α, and the corresponding weights have a posterior distribution centered on zero. Therefore, the features associated with these weights do not play a role in the prediction of the model, resulting in a sparse model (Yu et al., 2015).

To maximize the log-likelihood, an iterative training scheme was used: ➀ initialize the hyperparameters α and β; ➁ then calculate the hyperparameters m and Σ of the posterior distribution according to Equations (13) and (14); ➂ check the log likelihood or the convergence of the weight w; if the convergence criterion is not met, update the hyperparameters α and β according to Equations (17), (18) and (19), and then return to the second step; if the convergence criterion is met, then $\hat{\text{w}}=\text{m}$.

## Results and discussion

### Model parameter setting

In this study, two ResNet34 models were trained as the source network. In ImageNet-based source network 1, the torch version of the pretrained model was used. In lung cancer WSI-based source network 2, a stochastic gradient descent algorithm with momentum as the optimizer (initial learning rate of 0.01, momentum of 0.9, and weight decay of 10^−5^) was selected for training, the loss function was cross entropy, the batch size was 200, and the training round was set to 200 epochs. ResNet18 based on diverse branch blocks was used as the target network. In the training of the target network, a stochastic gradient descent algorithm with momentum was selected as the optimizer (the initial learning rate was set to 10^−4^, the momentum was set to 0.9, and the weight decay was set to 10^−5^), the batch size was set to 200, and the training round was set to 200 epochs. The hyperparameter ς for feature matching was set to 0.5, and *f*_∅_ and *g*_∅_ were trained using the Adam optimizer (initial learning rate of 10^−4^ and weight decay of 10^−4^). In this paper, the proposed method was implemented using the PyTorch framework and trained on an RTX 3090. For fairness, the training parameters of the comparison algorithm were consistent with the training parameters of the method in this paper. Since the target network of this study has 3,904 reparameterized convolution kernels, a total of 3,904 image features were extracted for each patient.

### Evaluation index

This study drew the receiver operating characteristic (ROC) curve and calculated the area under the curve (AUC), F1 score, precision, accuracy, sensitivity, and specificity to evaluate the performance of the model.

### Comparison with traditional methods

The method of this paper was compared with the clinical model (CM) (Feng et al., 2019) based on clinical features and CT findings, ResNet18 model without transfer learning strategy (ResNet18_nTL), ResNet34 model without transfer learning strategy (ResNet34_ nTL), fine-tuning ResNet18 model based on lung WSIs (FT_ResNet18_LW), fine-tuning ResNet18 model based on ImageNet (FT_ResNet18_ImageNet), fine-tuning ResNet34 model based on lung WSIs (FT_ ResNet34_LW), and fine-tuning ResNet34 model based on ImageNet (FT_ResNet34_ImageNet).

**FIGURE 7:**
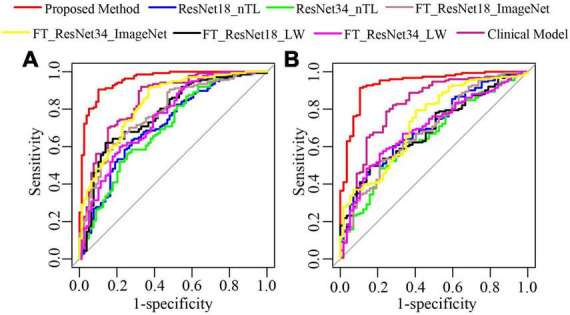
The ROC curves of the proposed method and traditional methods. **(A)** ROC of test cohort 1; **(B)** ROC of test cohort 2.

As shown in Figure 7, in test cohorts 1 and 2, the ROC of the proposed method was closest to the upper left corner of the image, which shows that the classification effect of the proposed method was more effective than the seven traditional methods. Table 2 presents the classification performance indicators of each model. The results of the proposed method (AUC: 0.9542 and 0.9356, F1 score: 0.9219 and 0.9356, and accuracy: 0.9028 and 0.9087) in the two test cohorts were higher than those of the seven traditional methods. The reason may be that the clinical model (AUC: 0.8485 and 0.8169) only uses the clinical features and CT findings for modeling and does not mine deep learning features. The proposed method conducted multifaceted mining of patient features, not only using deep learning features based on CT images but also using clinical features and CT findings for modeling. ResNet18_nTL and ResNet34_nTL were trained using only CT images of the training set, and the AUCs in the two test cohorts were only 0.7141 and 0.7066, 0.6981, and 0.6547, respectively. Due to the small number of CT images in the training set, the model was not adequately trained to the effect of the model in the two test sets was poor. FT_ResNet18_ImageNet and FT_ResNet34_ImageNet were obtained by using ImageNet as the source domain data to fine-tune ResNet18 and ResNet34, respectively. The AUCs of test cohort 1 were 0.7835 and 0.8303, respectively. The AUCs of test cohort 2 were 0.6901 and 0.7330, respectively. FT_ResNet18_LW and FT_ResNet34_LW were obtained by

**TABLE 2:** The performance indices of the proposed method and seven traditional models.

	Model	AUC (95 CI%)	F1 score	Precision	Sensitivity	Specificity	Accuracy
**Test Cohort 1**	Proposed method	**0.9542 (0.9265-0.9818)**	**0.9219**	**0.9394 (124/132)**	0.9051 (124/137)	**0.8987 (71/79)**	**0.9028 (195/216)**
	Clinical model	0.8485 (0.7932-0.9038)	0.8720	0.8289 (126/152)	**0.9197** (126/137)	0.6709 (53/79)	0.8287 (179/216)
	ResNet18 _nTL	0.7141 (0.6420-0.7863)	0.7073	0.7982 (87/109)	0.6350 (87/137)	0.7215 (57/79)	0.6667 (144/216)
	ResNet34 _nTL	0.6981 (0.6243-0.7720)	0.6581	0.7938 (77/97)	0.5620 (77/137)	0.7468 (59/79)	0.6296 (136/216)
	FT_ResNet18 _LW	0.7771 (0.7118-0.8425)	0.7296	0.8854 (85/96)	0.6204 (85/137)	0.8608 (68/79)	0.7083 (153/216)
	FT_ResNet18 _ImageNet	0.7835 (0.7211-0.8460)	0.7149	0.8571 (84/98)	0.6131 (84/137)	0.8228 (65/79)	0.6898 (149/216)
	FT_ResNet34 _LW	0.7567 (0.6890-0.8245)	0.6949	0.8283 (82/99)	0.5985 (82/137)	0.7848 (62/79)	0.6667 (144/216)
	FT_ResNet34 _ImageNet	0.8303 (0.7735-0.8870)	0.8591	0.8117 (125/154)	0.9124 (125/137)	0.6329 (50/79)	0.8102 (175/216)
**Test Cohort 2**	Proposed method	**0.9356 (0.8973-0.9740)**	0.**9356**	**0.9583 (138/144)**	**0.9139 (138/151)**	**0.8947 (51/57)**	**0.9087 (189/208)**
	Clinical model	0.8169 (0.7476-0.8861)	0.8380	0.8947 (119/133)	0.7881 (119/151)	0.7544 (43/57)	0.7788 (162/208)
	ResNet18 _nTL	0.7066 (0.6306-0.7827)	0.6383	0.8929 (75/84)	0.4967 (75/151)	0.8421 (48/57)	0.5913 (123/208)
	ResNet34 _nTL	0.6547 (0.5733-0.7361)	0.6186	0.8588 (73/85)	0.4834 (73/151)	0.7895 (45/57)	0.5673 (118/208)
	FT_ResNet18 _LW	0.6913 (0.6159-0.7667)	0.6383	0.8929 (75/84)	0.4967 (75/151)	0.8421 (48/57)	0.5913 (123/208)
	FT_ResNet18 _ImageNet	0.6901 (0.6101-0.7702)	0.6529	0.8681 (79/91)	0.5232 (79/151)	0.7895 (45/57)	0.5962 (124/208)
	FT_ResNet34 _LW	0.7006 (0.6244-0.7768)	0.6855	0.8763 (85/97)	0.5629 (85/151)	0.7895 (45/57)	0.6250 (130/208)
	FT_ResNet34 _ImageNet	0.7330 (0.6568-0.8092)	0.8163	0.8392 (120/143)	0.7947 (120/151)	0.5965 (34/57)	0.7440 (154/208)

**FIGURE 8:**
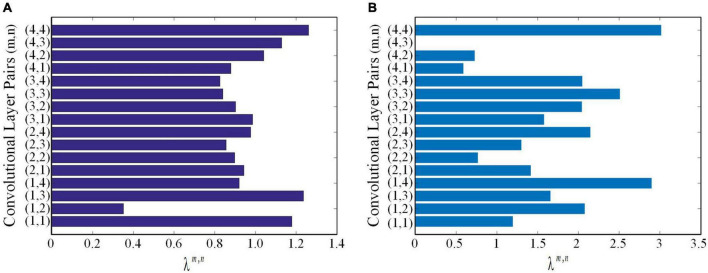
The matching weights ^λ*m*,*n*^ of convolutional layer pairs. **(A)** The matching weights ^λ*m*,*n*^ of convolutional layer pairs of the source network based on ImageNet. **(B)** The matching weights ^λ*m*,*n*^ of convolutional layer pairs of the source network based on lung WSIs.

Bold parts in the table represent the best performance. CI: confidence interval.

using lung WSIs as the source domain data to fine-tune ResNet18 and ResNet34, respectively. The AUCs of test cohort 1 were 0.7771 and 0.7567, respectively. The AUCs of test cohort 2 were 0.6913 and 0.7006, respectively. The results of the proposed method were all better than those of methods based on fine-tuning transfer learning. The reasons may be as follows: ➀ Compared with the method based on fine-tuning transfer learning, the proposed method had richer reference knowledge for training the target network. ➁ In this paper, the transfer weights were set for the features of the source network, in which the features of the two source networks that were beneficial to the learning of the target task were selectively restricted to the training of the target network. Figure 8 shows the matching weights ^λ*m*,*n*^ of the convolution layer pairs between the target network and source networks 1 and 2. It can be seen that different layer pairs of the source network and the target network had different matching weights. More weight was given to the source domain features with a positive effect on target network training. A smaller or even zero weight was given to the source domain features with a negative effect on target network training.

### Comparison with transfer learning models based on different source domains

**FIGURE 9:**
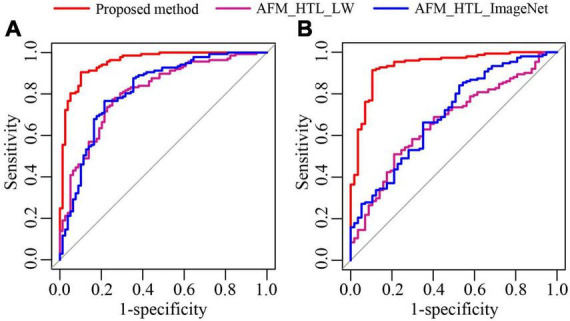
The ROC curves of the proposed method and transfer learning models based on different source domains. **(A)** ROC of test cohort 1; **(B)** ROC of test cohort 2.

To analyze the influence of the source domain data on transfer learning, the proposed method was compared with adaptive feature matching-based heterogeneous transfer learning based on lung WSIs (AFM_HTL_LW) and adaptive feature matching-based heterogeneous transfer learning based on ImageNet (AFM_HTL_ImageNet). The results are shown in Figure 9 and Table 3. In the two test cohorts, the AUCs of AFM_HTL_LW were 0.8103 and 0.6647, respectively; the F1 scores were 0.8061 and 0.6417, respectively; and the accuracies were 0.7639 and 0.5865, respectively. The AUCs of AFM_HTL_ImageNet were 0.8182 and 0.6983, respectively; the F1 scores were 0.8108 and 0.8427, respectively; and the accuracies were 0.7731 and 0.7404, respectively. It can be seen that the proposed method combined with the dual source domain outperforms the model based on a single source domain. This shows that the feature based on single source domain transfer learning was less robust than the features of transfer learning based on the dual-source domain. The proposed method used the effective knowledge of the two transfer sources to constrain the training of the target network so that the trained features were more relevant to the task and had better robustness.

**TABLE 3:** The performance indices of the proposed method and transfer learning models based on different source domains.

	Model	AUC (95 CI%)	F1 score	Precision	Sensitivity	Specificity	Accuracy
**Test Cohort 1**	Proposed method	**0.9542 (0.9265-0.9818)**	**0.9219**	**0.9394 (124/132)**	**0.9051 (124/137)**	**0.8987 (71/79)**	**0.9028 (195/216)**
	AFM_HTL_LW	0.8103 (0.7508-0.8699)	0.8061	0.8413 (106/126)	0.7737 (106/137)	0.7468 (59/79)	0.7639 (165/216)
	AFM_HTL_ ImageNet	0.8182 (0.7569-0.8795)	0.8108	0.8607 (105/122)	0.7664 (105/137)	0.7848 (62/79)	0.7731 (167/216)
**Test Cohort 2**	Proposed method	**0.9356 (0.8973-0.9740)**	**0.9356**	**0.9583 (138/144)**	**0.9139 (138/151)**	**0.8947 (51/57)**	**0.9087 (189/208)**
	AFM_HTL_LW	0.6647 (0.5848-0.7446)	0.6417	0.8652 (77/89)	0.5099 (77/151)	0.7895 (45/57)	0.5865 (122/208)
	AFM_HTL_ ImageNet	0.6983 (0.6184-0.7781)	0.8427	0.8089 (127/157)	0.8411 (127/151)	0.4737 (27/57)	0.7404 (154/208)

Bold parts in the table represent the best performance. CI: confidence interval.

### Ablation experiments

**FIGURE 10:**
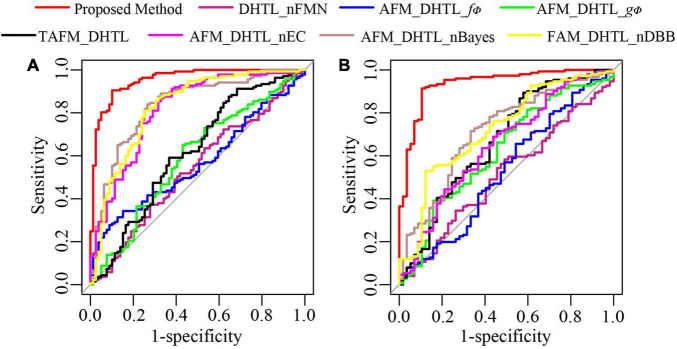
The ROC curves of the proposed method and ablation experiments. **(A)** ROC of test cohort 1; (**B**) ROC of test cohort 2.

To further demonstrate the performance of the proposed method, we conduct ablation experiments. As shown in Table 4, the dual-source domain heterogeneous transfer learning of the no-feature matching network (DHTL_nFMN) in which all source domain features are transferred to the target network, the adaptive feature matching-based dual-source domain heterogeneous transfer learning model based on *f*_∅_ (AFM_DHTL_*f*_∅_) that uses only *f*_∅_ for source domain feature selection, and the adaptive feature matching-based dual-source domain heterogeneous transfer learning based on *g*_Φ_ (AFM_DHTL_*g*_Φ_) that uses only *g*_Φ_ for source domain feature selection are compared with the proposed method. The results are shown in Figure 10 and Table 5. In the two test cohorts,

**TABLE 4:** Ablation experiment of the structure of the model.

Model	Diverse branch block	*f*_∅_	*g*_∅_	ELM	Sparse Bayes	Ensemble learning
Proposed method	**√**	**√**	**√**	**√**	**√**	**√**
DHTL_nFMN	**√**	×	×	**√**	**√**	**√**
AFM_DHTL_*f*_∅_	**√**	**√**	×	**√**	**√**	**√**
AFM_DHTL_*g*_Φ_	**√**	×	**√**	**√**	**√**	**√**
TAFM_DHTL	**√**	**√**	**√**	×	×	×
AFM_DHTL_nBayes	**√**	**√**	**√**	**√**	×	**√**
AFM_DHTL_nEC	**√**	**√**	**√**	**√**	**√**	×
AFM_DHTL_nDBB	×	**√**	**√**	**√**	**√**	**√**

√ means that the model has this structure; × means that the model does not have this structure.

**TABLE 5:** The performance indices of the proposed method and seven contrast models.

	Model	AUC (95 CI%)	F1 score	Precision	Sensitivity	Specificity	Accuracy
**Test Cohort 1**	Proposed method	**0.9542 (0.9265-0.9818)**	**0.9219**	**0.9394 (124/132)**	**0.9051 (124/137)**	**0.8987 (71/79)**	**0.9028 (195/216)**
	DHTL _nFMN	0.5371 (0.4568-0.6174)	0.6541	0.6744 (87/129)	0.6350 (87/137)	0.4684 (37/79)	0.5741 (124/216)
	AFM_ DHTL_*f*_∅_	0.5660 (0.4896-0.6424)	0.4796	0.7966 (47/59)	0.3431 (47/137)	0.8481 (67/79)	0.5278 (114/216)
	AFM_ DHTL_*g*_Φ_	0.5902 (0.5102-0.6703)	0.6846	0.7236 (89/123)	0.6496 (89/137)	0.5696 (45/79)	0.6204 (134/216)
	TAFM _DHTL	0.6164 (0.5355-0.6972)	0.7792	0.7018 (120/171)	0.8759 (120/137)	0.3544 (28/79)	0.6852 (148/216)
	AFM_DHTL_nBayes	0.8354 (0.7781-0.8928)	0.8425	0.8456 (115/136)	0.8394 (115/137)	0.7342 (58/79)	0.8009 (173/216)
	AFM_DHTL_nEC	0.8165 (0.7556-0.8775)	0.6440	0.8207 (119/145)	0.8686 (119/137)	0.6709 (53/79)	0.7963 (172/216)
	AFM_DHTL_nDBB	0.8261 (0.7651-0.8871)	0.8284	0.8473 (111/131)	0.8102 (111/137)	0.7468 (59/79)	0.7870 (170/216)
**Test Cohort 2**	Proposed method	**0.9356 (0.8973-0.9740)**	**0.9356**	**0.9583 (138/144)**	**0.9139 (138/151)**	**0.8947 (51/57)**	**0.9087 (189/208)**
	DHTL _nFMN	0.5095 (0.4226-0.5963)	0.6592	0.7586 (88/116)	0.5828 (88/151)	0.5088 (29/57)	0.5625 (117/208)
	AFM_ DHTL_*f*_∅_	0.5257 (0.4340-0.6175)	0.7046	0.7615 (99/130)	0.6556 (99/151)	0.4561 (26/57)	0.6010 (125/208)
	AFM_ DHTL_*g*_Φ_	0.6225 (0.5354-0.7096)	0.5388	0.8676 (59/68)	0.3907 (59/151)	0.8421 (48/57)	0.5144 (107/208)
	TAFM _DHTL	0.6681 (0.5803-0.7559)	0.8464	0.8036 (135/168)	0.8940 (135/151)	0.4211 (24/57)	0.7644 (159/208)
	AFM_DHTL_nBayes	0.7130 (0.6339-0.7922)	0.7770	0.8504 (108/127)	0.7152 (108/151)	0.6667 (38/57)	0.7019 (146/208)
	AFM_DHTL_nEC	0.6490 (0.5635-0.7345)	0.7429	0.8062 (104/129)	0.6887 (104/151)	0.5614 (32/57)	0.6538 (136/208)
	AFM_DHTL_nDBB	0.7235 (0.6452-0.8017)	0.6723	0.9195 (80/87)	0.5298 (80/151)	0.8772 (50/57)	0.6250 (130/208)

Bold parts in the table represent the best performance. CI: confidence interval.

the AUCs of DHTL_nFMN were 0.5371 and 0.5095, the AUCs of AFM_DHTL_*f*_∅_ were 0.5660 and 0.5257, and the AUCs of AFM_DHTL_*g*_Φ_ were 0.5902 and 0.6225, respectively. The results of the proposed method were better than the results of the three comparison models mentioned above. The results showed that when using transfer learning to train the target network, choosing the appropriate transfer configuration has a greater impact on the performance of the model. The results also showed that the proposed method can automatically determine useful feature pairs from many possible candidate transfer pairs to constrain the training of the target network and improve the robustness of the characteristics of the target network.

Compared with the proposed method, the results of the traditional adaptive feature matching-based dual-source domain heterogeneous transfer learning (TAFM_DHTL) were directly given by the fully connected layer of the target network ResNet18. The adaptive feature matching based dual-source domain heterogeneous transfer learning of non-Bayes (AFM_DHTL_nBayes) with the ensemble classifier of traditional ELM as the final classifier was constructed. The adaptive feature matching-based dual-source domain heterogeneous transfer learning with a non-ensemble classifier (AFM_DHTL_nEC) that used sparse Bayesian ELM as the final classifier was constructed. In the two test cohorts, the AUCs of TAFM_DHTLM were 0.6164 and 0.6681, the AUCs of AFM_DHTL_nBayes were 0.8354 and 0.7130, and the AUCs of AFM_DHTL_nEC were 0.8165 and 0.6490, respectively. The results of the above three comparison models were all worse than the results of the proposed method. The reasons may be that TAFM_HTL was directly given by the fully connected layer of the target network ResNet18, and the results may be affected by redundant features, thus reducing the performance of the model. AFM_DHTL_nBayes used the ensemble classifier based on traditional ELM, which does not have the ability of superior feature selection and base classifier selection. AFM_DHTL_nEC used the output of a single sparse Bayesian ELM model as the final output, which does not have the advantages of ensemble learning to brainstorm. The proposed method not only comprehensively analyzes the features in the target network but also uses the sparse Bayes-based ELM ensemble classifier to screen and classify the features and filter out the base classifiers that do not work or play a negative role in ensemble learning.

The adaptive feature matching-based dual-source domain heterogeneous transfer learning with non- diverse branch block (AFM_DHTL_nDBB) that used the traditional ResNet18 was constructed and compared with the proposed method. In the two test cohorts, the AUCs of AFM_DHTL_nDBB were 0.8261 and 0.7235, respectively. Because the proposed method introduces the diverse branch block structure into the target network, the target network can train more robust features, thereby improving the robustness of the classification model.

## Conclusion

Aiming at the problem of negative transfer of redundant features when the features of the source network are transferred to the target network as a whole in heterogeneous transfer learning, according the mechanism of the human brain focusing on effective knowledges while ignoring redundant knowledges in recognition tasks, a brain-like classification method for CT images based on adaptive feature matching dual-source domain heterogeneous transfer learning was proposed for the preoperative differentiation of LG and LA appearing as SPSN. The method can adaptively select the features in the source network that are conducive to the learning of the target task and the destination of the feature transfer by designing an adaptively selected feature matching network. Thus, the training of the target network was constrained, and the robustness of the target network was improved in the case of small samples. At the same time, a target network based on diverse branch block was proposed, which makes the target network have different receptive fields and complex paths and further improves the feature expression ability of the target network. In addition, the clinical features and CT findings were included to analyze and conduct a comprehensive analysis of the patients. After that, an ensemble classifier based on sparse Bayesian ELM was proposed to automatically combine the outputs of the base classifiers to improve the classification performance. Finally, experiments on the data of two medical centers verify the effectiveness of our method (test cohort 1 AUC: 0.9542 and test cohort 2 AUC: 0.9356). In future work, we intend to explore how to introduce Bayesian theory into heterogeneous transfer learning and use the uncertainty of Bayesian theory to improve the accuracy of the model in the case of small samples.

## Data availability statement

The raw data supporting the conclusions of this article will be made available by the authors, without undue reservation.

## Ethics statement

The studies involving human participants were reviewed and approved by Ethics Committee of Jiangmen Central Hospital (approval number: [2020]41). The ethics committee waived the requirement of written informed consent for participation.

## Author contributions

YC and XC contributed to the conception and design of the study. XC organized the database and wrote sections of the manuscript. YC performed the data analysis and wrote the first draft of the manuscript. Both authors read and agreed to the published version of the manuscript.
